# AMP‐activated protein kinase inhibits K_v_1.5 channel currents of pulmonary arterial myocytes in response to hypoxia and inhibition of mitochondrial oxidative phosphorylation

**DOI:** 10.1113/JP272032

**Published:** 2016-06-30

**Authors:** Javier Moral‐Sanz, Amira D. Mahmoud, Fiona A. Ross, Jodene Eldstrom, David Fedida, D. Grahame Hardie, A. Mark Evans

**Affiliations:** ^1^ Centre for Integrative PhysiologyCollege of Medicine and Veterinary MedicineHugh Robson BuildingGeorge SquareUniversity of EdinburghEdinburghEH8 9XDUK; ^2^ Division of Cell Signalling & ImmunologySchool of Life SciencesUniversity of DundeeDow StreetDundeeDD1 5EHUK; ^3^ Department of Anaesthesiology. Pharmacology and TherapeuticsUniversity of British Columbia2350 Health Science MallVancouverCanadaV6T 1Z3

## Abstract

**Key points:**

- Progression of hypoxic pulmonary hypertension is thought to be due, in part, to suppression of voltage‐gated potassium channels (K_v_) in pulmonary arterial smooth muscle by hypoxia, although the precise molecular mechanisms have been unclear.
- AMP‐activated protein kinase (AMPK) has been proposed to couple inhibition of mitochondrial metabolism by hypoxia to acute hypoxic pulmonary vasoconstriction and progression of pulmonary hypertension.
- Inhibition of complex I of the mitochondrial electron transport chain activated AMPK and inhibited K_v_1.5 channels in pulmonary arterial myocytes.
- AMPK activation by 5‐aminoimidazole‐4‐carboxamide riboside, A769662 or C13 attenuated K_v_1.5 currents in pulmonary arterial myocytes, and this effect was non‐additive with respect to K_v_1.5 inhibition by hypoxia and mitochondrial poisons.
- Recombinant AMPK phosphorylated recombinant human K_v_1.5 channels in cell‐free assays, and inhibited K^+^ currents when introduced into HEK 293 cells stably expressing K_v_1.5.
- These results suggest that AMPK is the primary mediator of reductions in K_v_1.5 channels following inhibition of mitochondrial oxidative phosphorylation during hypoxia and by mitochondrial poisons.

**Abstract:**

Progression of hypoxic pulmonary hypertension is thought to be due, in part, to suppression of voltage‐gated potassium channels (K_v_) in pulmonary arterial smooth muscle cells that is mediated by the inhibition of mitochondrial oxidative phosphorylation. We sought to determine the role in this process of the AMP‐activated protein kinase (AMPK), which is intimately coupled to mitochondrial function due to its activation by LKB1‐dependent phosphorylation in response to increases in the cellular AMP:ATP and/or ADP:ATP ratios. Inhibition of complex I of the mitochondrial electron transport chain using phenformin activated AMPK and inhibited K_v_ currents in pulmonary arterial myocytes, consistent with previously reported effects of mitochondrial inhibitors. Myocyte K_v_ currents were also markedly inhibited upon AMPK activation by A769662, 5‐aminoimidazole‐4‐carboxamide riboside and C13 and by intracellular dialysis from a patch‐pipette of activated (thiophosphorylated) recombinant AMPK heterotrimers (α2β2γ1 or α1β1γ1). Hypoxia and inhibitors of mitochondrial oxidative phosphorylation reduced AMPK‐sensitive K^+^ currents, which were also blocked by the selective K_v_1.5 channel inhibitor diphenyl phosphine oxide‐1 but unaffected by the presence of the BK_Ca_ channel blocker paxilline. Moreover, recombinant human K_v_1.5 channels were phosphorylated by AMPK in cell‐free assays, and K^+^ currents carried by K_v_1.5 stably expressed in HEK 293 cells were inhibited by intracellular dialysis of AMPK heterotrimers and by A769662, the effects of which were blocked by compound C. We conclude that AMPK mediates K_v_ channel inhibition by hypoxia in pulmonary arterial myocytes, at least in part, through phosphorylation of K_v_1.5 and/or an associated protein.

## Key points

- Progression of hypoxic pulmonary hypertension is thought to be due, in part, to suppression of voltage‐gated potassium channels (K_v_) in pulmonary arterial smooth muscle by hypoxia, although the precise molecular mechanisms have been unclear.
- AMP‐activated protein kinase (AMPK) has been proposed to couple inhibition of mitochondrial metabolism by hypoxia to acute hypoxic pulmonary vasoconstriction and progression of pulmonary hypertension.
- Inhibition of complex I of the mitochondrial electron transport chain activated AMPK and inhibited K_v_1.5 channels in pulmonary arterial myocytes.
- AMPK activation by 5‐aminoimidazole‐4‐carboxamide riboside, A769662 or C13 attenuated K_v_1.5 currents in pulmonary arterial myocytes, and this effect was non‐additive with respect to K_v_1.5 inhibition by hypoxia and mitochondrial poisons.
- Recombinant AMPK phosphorylated recombinant human K_v_1.5 channels in cell‐free assays, and inhibited K^+^ currents when introduced into HEK 293 cells stably expressing K_v_1.5.
- These results suggest that AMPK is the primary mediator of reductions in K_v_1.5 channels following inhibition of mitochondrial oxidative phosphorylation during hypoxia and by mitochondrial poisons.

**Abbreviations**
AICAR: 5‐aminoimidazole‐4‐carboxamide riboside
AMPK: AMP‐activated protein kinase
BK_Ca_: large conductance voltage‐ and calcium‐activated K^+^ channel
DPO‐1: diphenyl phosphine oxide‐1
HEK 293: human embryonic kidney 293 cells
HPV: hypoxic pulmonary vasoconstriction
K_v_: voltage‐gated K^+^ channel
LKB1: liver kinase B1

## Introduction

Hypoxia without hypercapnia induces pulmonary vasoconstriction, and thus assists ventilation–perfusion matching in the lung (von Euler & Liljestrand, 1946). However, hypoxia may trigger pulmonary hypertension when it is widespread, for example during ascent to altitude (Bartsch *et al*. 2005) or due to disorders such as cystic fibrosis (Lahm *et al*. 2014). While current therapies have been shown to prolong survival, pulmonary hypertension remains a life‐threatening disorder (Lahm *et al*. 2014) and the precise molecular mechanisms underlying it remain unclear. Therefore, greater understanding is critical to the development of effective therapies.

Initially, hypoxic pulmonary vasoconstriction (HPV) is driven by calcium release via ryanodine receptors from the sarcoplasmic reticulum of pulmonary arterial smooth muscle cells (Dipp *et al*. 2001), but is also associated with concomitant inhibition of voltage‐gated potassium channels (K_v_) (Post *et al*. 1992; Yuan *et al*. 1993; Archer *et al*. 2004). The role of K_v_ channel inhibition in acute HPV remains open to debate (Wilson *et al*. 2002; Wang *et al*. 2004; Evans *et al*. 2005; Lu *et al*. 2008), but it has been proposed that loss of K_v_ function contributes to smooth muscle proliferation and thus to the progression of pulmonary hypertension (Sweeney & Yuan, 2000; Moudgil *et al*. 2006) by promoting cell survival (Ekhterae *et al*. 2001, 2003).

K_v_ current suppression during hypoxia (Post *et al*. 1992; Yuan *et al*. 1993; Firth *et al*. 2008) occurs as a consequence of inhibition of mitochondrial oxidative phosphorylation (Firth *et al*. 2008, 2009). However, the nature of the signalling pathway that couples mitochondrial function to K_v_ channels has been unclear. In this respect, little attention has been paid to the role of the AMP‐activated protein kinase (AMPK), although we have previously proposed that it couples inhibition of mitochondrial metabolism by hypoxia to acute HPV (Evans *et al*. 2005; Evans, 2006) and may also contribute to the progression of pulmonary hypertension (Evans *et al*. 2005; Evans, 2006; Ibe *et al*. 2013; Goncharov *et al*. 2014). AMPK, an energy sensor that acts to maintain cellular energy homeostasis, exists as heterotrimers comprising catalytic α subunits and regulatory β and γ subunits (Hardie, 2014a,b,c). AMPK is coupled to mitochondrial metabolism through changes in the cellular AMP:ATP and ADP:ATP ratios. Binding of AMP to the γ subunit causes a 10‐fold increase in AMPK activity by allosteric activation, but a further activation of up to 100‐fold can be generated by binding of either AMP or ADP, which promotes phosphorylation and inhibits dephosphorylation of Thr172 on the α subunit; these effects are antagonised by ATP (Gowans *et al*. 2013; Ross *et al*. 2016). Thr172 is primarily phosphorylated by the tumour suppressor kinase LKB1 (liver kinase B1), which appears to be constitutively active (Sakamoto *et al*. 2004), but which phosphorylates AMPK more rapidly when AMP is bound to the γ subunit. In an alternative Ca^2+^‐dependent activation mechanism, the calmodulin‐dependent protein kinase CaMKKβ can also phosphorylate Thr172 and hence activate AMPK in an AMP‐independent manner (Hardie, 2014a,b,c). The classical role of AMPK is to maintain energy homeostasis under conditions of metabolic stress, by activating catabolic processes that generate ATP and inhibiting non‐essential anabolic processes that consume ATP. However, AMPK has also been shown to regulate a wide variety of ion channels and membrane transport proteins (Evans *et al*. 2009; Lang & Foller, 2014), including K_v_2.1 (Ikematsu *et al*. 2011), KCa3.1 (Ross *et al*. 2011), and Kir 2.1 and K_v_7.1 (Lang & Foller, 2014).

Of the various known K_v_ channel types, it has been established that both K_v_2.1 and K_v_1.5 contribute to voltage‐gated potassium currents in pulmonary arterial myocytes (Smirnov *et al*. 2002; Archer *et al*. 2004; Firth *et al*. 2011; Olschewski *et al*. 2014). Their relative contributions vary in a manner related to arterial diameter, with the greatest level of K_v_1.5 expression (and contribution to K_v_ currents) occurring in myocytes from near‐resistance‐sized arteries (Archer *et al*. 1998, 2004; Smirnov *et al*. 2002; Moral‐Sanz *et al*. 2011), the response of which to hypoxia is critical to acute increases in pulmonary arterial perfusion pressure. Moreover, selective down‐regulation of K_v_1.5 has been identified as a hallmark of pulmonary hypertension (Yuan *et al*. 1998; Michelakis *et al*. 2002; Bonnet *et al*. 2006; Remillard *et al*. 2007; Burg *et al*. 2010; Morales‐Cano *et al*. 2014). Consistent with this view, overexpression of K_v_1.5 enhances apoptosis (Brevnova *et al*. 2004), while adenoviral expression of a K_v_1.5 transgene *in vivo* reduces pulmonary hypertension and restores HPV (Pozeg *et al*. 2003).

We show here that AMPK selectively inhibits K_v_1.5 in pulmonary arterial myocytes, and also phosphorylates and inhibits recombinant K_v_1.5 channels expressed in HEK 293 cells.

## Methods

### Ethical approval

All experiments were performed under the United Kingdom Animals (Scientific Procedures) Act 1986. The animals used in this study were male Sprague Dawley rats that underwent no experimental procedures as recognised under UK Law. They were killed using a Schedule 1 method for collection of tissues only, which does not require formal ethical approval in the UK. Frozen canine tissue was left over from a previous project where surgical procedures and protocols were approved by the Cleveland Clinic Foundation Institutional Animal Care and Use Committee (Cleveland, OH, USA).

### Smooth muscle cell isolation

Resistance pulmonary arteries (<200 μm inner diameter) from male Sprague Dawley rats (250–350 g) were dissected into a physiological bath solution of composition (in mm): NaCl 135, KCl 5, MgCl_2_ 1, CaCl_2_ 1, glucose 10, Hepes 10 (pH 7.4). For cell isolation, endothelium denuded arteries were transferred into a nominally calcium‐free bath solution containing (in mg ml^−1^): 1 papain, 0.8 dithiothreitol and 0.7 BSA. The tissue was incubated in the latter solution for 10 min at 37°C and gently triturated using a fire polished glass pipette to get dispersed pulmonary arterial smooth muscle cells.

### Electrophysiological recordings

Pulmonary arterial myocytes or HEK 293 cells that stably expressed K_v_1.5 were transferred to a recording chamber and perfused at 1 ml min^−1^ with bath solution. K^+^ currents were recorded by whole‐cell patch clamp and a pipette solution of the following composition (mm): KCl 140, MgCl_2_ 1, EGTA 10, Hepes 10, Na_2_ATP 4, Na_3_GTP 0.1 (pH 7.2). Cells were superfused (3 ml min^−1^) at 37°C with bath solution steadily bubbled with either room air (normoxia) or 95% N_2_/5% CO_2_ [hypoxia, 4.4 ± 0.3% O_2_ in the experimental chamber; as measured with an optical oxygen meter (FireStingO2, Pyro Science, Aachen, Germany)]. For some experiments recombinant thiophosphorylated AMPK heterotrimers (α2β2γ1, α1β1γ1 or D157A kinase dead mutant) were added to the pipette solution. K_v_ currents were assessed by voltage ramps (−100 to +40 mV), single voltage steps (−80 to +40 mV) and by acquisition of full *I–V* relationships for steady state activation (200 ms steps from −80 to +40 mV in 10 mV increments) or inactivation (2 s inactivation steps from −80 to +40 mV in 10 mV increments, a 10 ms pre‐pulse at −80 mV followed by a single voltage step to +60 mV). Current magnitude was normalised to cell capacitance as required. Conductance values (*G*) were calculated from the equation *G* = *I*/(*V* − EK), where the Nernst equilibrium potential (EK) was calculated as −89 mV at 37°C. Normalised conductance/voltage profiles for K_v_ currents were fitted to a single Boltzmann function with the form *G* = *G*_max_/(1 + exp[− (*V* − *V*_mid_)/*k*]), where *G*_max_ is the maximal conductance, *V*_mid_ is the test potential for half‐maximal conductance (*G*_0.5_) and *k* represents the slope of the activation curve. Patch pipettes had resistances of 4–6 MΩ. Series resistance was compensated for (60–80%) after achieving the whole‐cell configuration. Signals were sampled at 10 kHz and low‐pass filtered at 2 kHz. Voltage‐clamp acquisition and analysis protocols were performed using an Axopatch 200A amplifier/Digidata 1200 interface controlled by Clampex 10.0 software (Molecular Devices, Sunnyvale, CA, USA). Off‐line analysis was performed using Clampfit 10.0 (Molecular Devices). Data are expressed as current density (pA pF^–1^) or *I*/*I*_zero_, where *I*_zero_ is the current magnitude recorded at the onset of a given experimental intervention.

### Cell culture and transfection

HEK 293 cells were cultured in Dulbecco's modified Eagle's medium (DMEM) supplemented with 10% (v/v) fetal bovine serum and 1% (v/v) penicillin/streptomycin. Cells were transfected following the manufacturer's instructions with 12 μg of pcDNA3.1 encoding an HA‐tagged human K_v_1.5 (KCNA5) using Fugene 6 (Promega, Madison, WI, USA) and lysed 48 h later.

### RT‐PCR

Total RNA was isolated from frozen canine tissues and from frozen pelleted HEK 293 cells stably expressing K_v_1.5, using the RNeasy Mini Kit (Qiagen, Valencia, CA, USA) as per the manufacturer's instructions. Reverse transcription PCRs (RT‐PCRs) were carried out on 200 ng of total RNA using the One‐Step RT‐PCR Kit (ABM, Richmond, Canada) as recommended in the manual. Then, 20 μl of the 50 μl reaction was run on a 1% agarose gel and visualised using Safe‐White (ABM) and a GelDoc equipped with Quantity One software (BioRad, Hercules, CA, USA). K_v_β primer sequences were as previously published (Platoshyn *et al*. 2004) and for the reference gene ReadyMade GAPDH primers (Integrated DNA Technologies, Coralville, IA, USA) were used with sequences as follows: GAPDH‐For, ACCACAGTCCATGCCATCAC; GAPDH‐Rev, TCCACCACCCTGTTGCTGTA. RT‐PCR was repeated with 250 ng of template and 35 cycles of PCR after very faint bands were observed in the K_v_1.5 stable cell line for K_v_β1 and K_v_β3. Canine ventricle was also repeated for comparison.

### K_v_1.5 phosphorylation assays

Phosphorylation assays were performed as described previously (Ross *et al*. 2011) using AMPK purified from bacteria and phosphorylated with CAMKKβ (10 units ml^−1^) in the presence of 200 μm AMP for 30 min at 30°C.

### Expression, purification and activation of bacterial AMPK

These were performed as described previously (Ross *et al*. 2011).

### Isoform‐specific AMPK activities

Isoform‐specific AMPK activity was determined by immunoprecipitating tissue lysate with antibodies raised against α1 or α2 subunits bound to protein G‐Sepharose beads and quantified using the AMARA peptide and [γ‐^32^P]ATP substrates (Cheung *et al*. 2000).

### Statistics

Data are expressed as means ± SEM or means ± SD, as indicated; *n* represents the number of cells tested from at least four different animals. Statistical analysis was performed using Student's *t* test for paired observations or one‐way ANOVA followed by a Dunnett's *post hoc* test. Differences were considered statistically significant at *P* < 0.05.

## Results

### Inhibition of mitochondrial oxidative phosphorylation activates AMPK and reduces K_v_1.5 current density in pulmonary arterial myocytes

**Figure 1:**
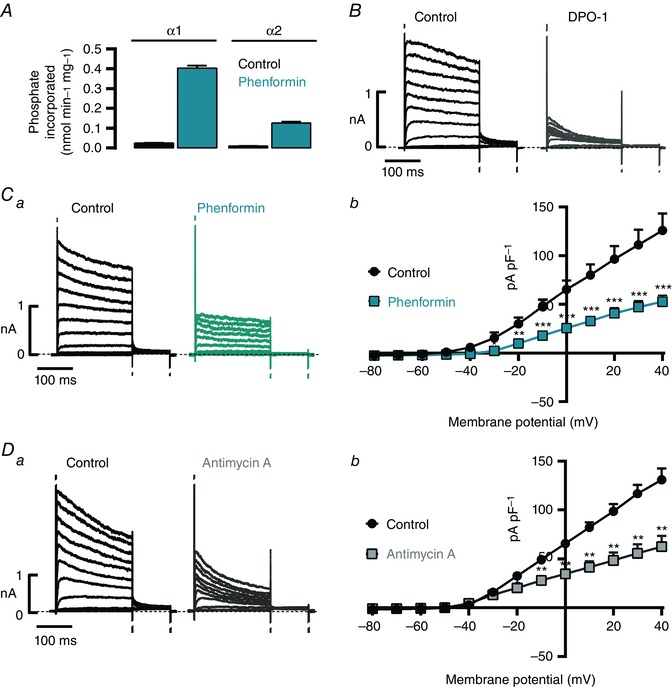
**Inhibition of mitochondrial oxidative phosphorylation activates AMPK and reduces K_v_1.5 current density in pulmonary arterial myocytes** *A*, bar chart showing the effect of 10 mm phenformin on the activity of AMPK‐α1 and AMPK‐α2 containing heterotrimers, as determined by immunoprecipitate kinase assay (*n* = 3; 32 arteries, 8 rats). *B*, representative records (200 ms pulses from −80 to +40 mV in 10 mV increments, holding potential −80 mV) in pulmonary arterial myocytes before (control) and after extracellular application of 1 μm DPO‐1. *C* and *D*, representative records (*a*) and associated *I–V* relationships (*b*; 200 ms depolarization pulses from −80 to +40 mV in 10 mV increments, holding potential −80 mV) from myocytes pre‐incubated with 1 mm phenformin *versus* time‐matched controls (*C*, green, *n* = 9–11), or before and after 5–8 min extracellular application of 1 μm antimycin A (*D*, *n* = 6). ^*^ *P* < 0.05, ^**^ *P* < 0.01 and ^***^ *P* < 0.001.

Biguanide drugs such as phenformin inhibit complex I of the mitochondrial respiratory chain (El‐Mir *et al*. 2000; Owen *et al*. 2000; Evans *et al*. 2005) and elicit consequent increases in the cellular AMP:ATP ratio and AMPK activation (Hawley *et al*. 2010). Consistent with this, pre‐incubation of second‐ and third‐order pulmonary arteries with phenformin (4 h) increased AMPK‐α1‐associated activity from (mean ± S.D.) 0.025 ± 0.001 to 0.403 ± 0.012 nmol min^−1^ mg^−1^ protein and AMPK‐α2‐associated activity from 0.0096 ± 0.001 to 0.126 ± 0.006 nmol min^−1^ mg^−1^ protein (Fig. 1 *A*, *n* = 3; 32 arteries, 8 rats). Furthermore, and in accordance with previously reported effects of mitochondrial inhibitors (Firth *et al*. 2008) and hypoxia (Platoshyn *et al*. 2001), pre‐incubation of acutely isolated pulmonary arterial myocytes with 1 mm phenformin (2–4 h) also caused pronounced reductions in K_v_ current density, from 126 ± 17 pA pF^–1^ in time‐matched controls to 55 ± 6 pA pF^–1^ at +40 mV (Fig. 1 *Ca–b*, *n* = 9–11, *P* < 0.001). Consistent with the effects of phenformin and previous investigations by others (Firth *et al*. 2008), acute application of antimycin A (1 μm), a rapidly acting inhibitor of complex III, caused equivalent reductions in K_v_ current density, from 131.3 ± 10.4 to 62.6 ± 11.1 pA pF^–1^ at +40 mV (Fig. 1 *Da–b*, *n* = 6, *P* < 0.001). Unless stated, in these and all subsequent experiments on pulmonary arterial myocytes, potassium currents were recorded in the presence of paxilline (1 μm) to block the large conductance voltage‐ and calcium‐activated K^+^ (BK_Ca_) channel.

Given that the contribution to native K_v_ currents of K_v_2.1 and K_v_1.5 varies, in a manner related to both the size and the regional location within the lung of the arteries from which myocytes are derived (Smirnov *et al*. 2002), we assessed the nature of the channels that underpin the K_v_ current within the cells under study. Application of the K_v_1.5 blocker diphenyl phosphine oxide‐1 (DPO‐1, 1 μm, Fig. 1 *B*) in the absence of paxilline caused almost total inhibition of the K_v_ currents (96 ± 1% at +40 mV; *n* = 6, *P* < 0.0001), consistent with the view that K_v_1.5 drives the majority of voltage‐gated K^+^ currents in myocytes of the near‐resistance‐sized pulmonary arteries studied here, which contribute most to the increase in pulmonary vascular resistance during hypoxia (Kato & Staub, 1966; Archer *et al*. 2004).

### AMPK activation inhibits K_v_1.5 currents in pulmonary arterial myocytes

**Figure 2:**
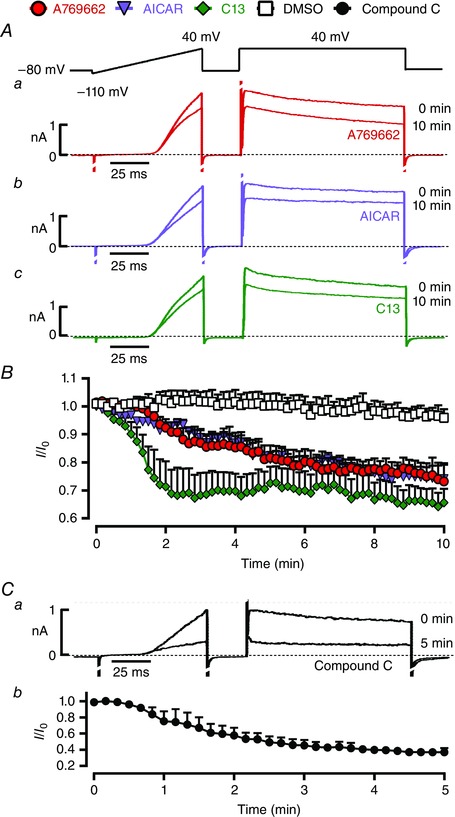
**Time course for reduction in K_v_ current in response to AMPK activators** *A*, representative current records for voltage ramp and step protocol at 0 and 10 min after extracellular application of 100 μm A769662 (*a*), 1 mm AICAR (*b*) or 30 μm C13 (*c*). *B*, time course for reductions in K_v_ current during 10 min of extracellular application of DMSO (1:1000, vehicle control), 100 μm A769662, 1 mm AICAR and 30 μm C13. *C*, the effect on K_v_ currents of pulmonary arterial myocytes of 5 min extracellular application of 30 μm compound C (*a*), and the time course for inhibition (*b*). Results are expressed as mean ± SEM, *n* = 3–8.

We next assessed the effect on K_v_1.5 current amplitude of extracellular application of three AMPK activators with distinct mechanisms of action, i.e. A769662, 5‐aminoimidazole‐4‐carboxamide riboside (AICAR) and C13. Analysis of the time course for K_v_1.5 inhibition at steady‐state activation (100 ms at +40 mV) showed the time to onset of effect for A769662 (100 μm), AICAR (1 mm) and C13 (30 μm) to be around 2 min, with apparent maxima for inhibition achieved after 8–10 min (Fig. 2 *B*). After 10 min, K_v_1.5 currents had declined (Fig. 2 *A*) from 144 ± 12 to 101 ± 9 pA pF^–1^ in the presence of A769662 (*n* = 14), from 186 ± 23 to 136 ± 17 pA pF^–1^ in the presence of AICAR (*n* = 6) and from 164 ± 8 to 104 ± 10 pA pF^–1^ in the presence of C13 (*n* = 8). Note, however, that in 3 of 9 cells superfused with AICAR we observed no effect (excluded from analysis).

Surprisingly, we also observed inhibition of K_v_1.5 currents upon application of the non‐selective AMPK antagonist compound C (30 μm), from 164 ± 62 to 59 ± 24 pA pF^–1^ (*n* = 3; Fig. 2 *C*); this confounding effect precluded the use of this agent in further studies on myocytes. Previous studies have shown that compound C has little or no effect on resting pulmonary arterial tone, but inhibits acute HPV in a concentration‐dependent manner (Robertson *et al*. 2008), which must therefore be induced independently of the inhibition by hypoxia of K_v_1.5 inhibition (Dipp *et al*. 2001). It is worth noting that in a screen of 70 protein kinases, at least 10 were inhibited by compound C more potently than AMPK (Bain *et al*. 2007). Thus, it is not a specific inhibitor of AMPK, a point reinforced by our findings that it markedly attenuates K_v_1.5 function. This should be considered when interpreting outcomes of other cell‐based assays that have employed compound C, not least with respect to myocyte proliferation and survival (Ibe *et al*. 2013).

**Figure 3:**
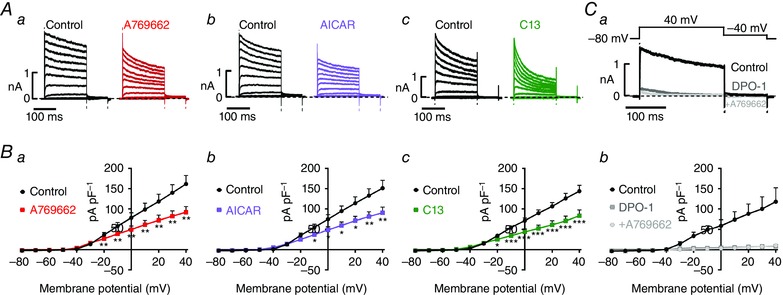
**AMPK activation inhibits K_v_1.5 currents in pulmonary arterial myocytes** *A*, representative current traces (200 ms depolarization pulses from −80 to +40 mV in 10 mV increments, holding potential −80 mV); and *B*, *I–V* relationships for K_v_ current recorded before (control) and after extracellular application of A769662 (*a*, 100 μm), AICAR (*b*, 1 mm) or C13 (*c*, 30 μm); measurements taken at end of pulse. *C*, representative current trances (*a*) and *I–V* relationships (*b*) for K_v_ currents in the absence and presence of 1 μm DPO‐1 and the effect of 100 μm A769662 in the continued presence of DPO‐1. Results are expressed as mean ± SEM, *n* = 5–7. ^*^ *P* < 0.05, ^**^ *P* < 0.01 and ^***^ *P* < 0.001.

Importantly, upon equilibration of pulmonary arterial myocytes with AICAR, A769662 and C13, reductions in current density were evident throughout the *I–V* range over which K_v_1.5 currents were activated (Fig. 3 *A*, *B*). This was confirmed by the fact that A769662 was without effect on residual currents observed following pre‐incubation of cells with DPO‐1 even in the absence of paxilline (Fig. 3 *C*), current density measuring 10 ± 2 pA pF^–1^ in the presence of DPO‐1 alone and 11 ± 3 pA pF^–1^ in the presence of DPO‐1 and A769662 (*n* = 6).

### Intracellular application of active AMPK heterotrimers inhibits K_v_1.5 in pulmonary arterial myocytes

**Figure 4:**
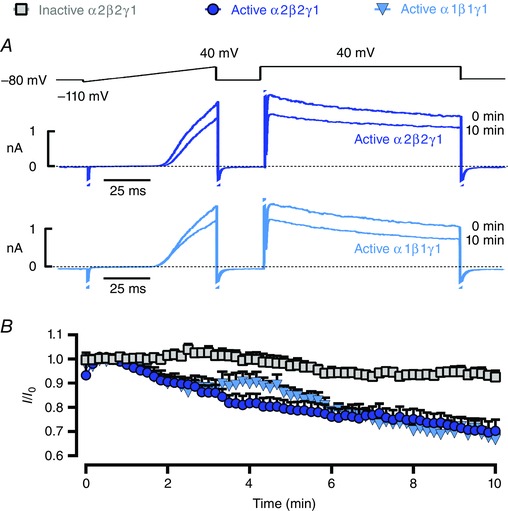
**Intracellular application of active AMPK heterotrimers inhibits K_v_1.5 in pulmonary arterial myocytes** *A*, voltage ramp and step protocol recorded at 0 and 10 min after intracellular dialysis of the indicated recombinant, thiophosphorylated active AMPK heterotrimers (α2β2γ1 or α1β1γ1). *B*, time course for reduction in K_v_ current following intracellular dialysis of either active α2β2γ1 (thiophosphorylated, 5 U ml^−1^), active α1β1γ1 (thiophosphorylated, 5 U ml^−1^) or inactive α2β2γ1 (D157A mutant) AMPK heterotrimer. Results are expressed as mean ± SEM, *n* = 5–7.

Although A769662, AICAR and C13 activate AMPK by different mechanisms and are therefore unlikely to have the same off‐target effects, we also analysed the effect on endogenous K_v_1.5 channel function of bacterially expressed human AMPK heterotrimers (α2β2γ1 or α1β1γ1 complexes). These had been thiophosphorylated at Thr172 using CaMKKβ to yield active, recombinant AMPK that is also resistant to phosphatases (Ross *et al*. 2011). Intracellular dialysis of either of the active α2β2γ1 or α1β1γ1 heterotrimers (Fig. 4 *A*) evoked K_v_1.5 current inhibitions that were similar in magnitude (−33 ± 5% for α2β2γ1 and −36 ± 7% for α1β1γ1 at +40 mV; *n* = 5–7) to the reductions induced by pharmacological activation of AMPK. Importantly, current inhibition was not observed upon intracellular dialysis of an inactive AMPK heterotrimer [Fig. 4 *B*; α2β2γ1 complex with D157A mutation in α2 (Ross *et al*. 2011)]. Based on the use both of pharmacological activation of endogenous AMPK and of exogenous recombinant AMPK, we can conclude that AMPK mediates, either directly or indirectly, inhibition of K_v_1.5 currents in pulmonary arterial myocytes.

### Hypoxia and mitochondrial inhibitors attenuate K_v_1.5 currents and occlude further current inhibition by AMPK activation

AMPK is intimately coupled to mitochondrial metabolism through changes in the AMP:ATP and ADP:ATP ratios (Gowans *et al*. 2013), which is evident from the fact that AMPK activity was increased by hypoxia (Evans *et al*. 2005) and by the mitochondrial inhibitor phenformin (Fig. 1 *A*). To assess whether AMPK acted as a downstream mediator of K_v_1.5 inhibition during hypoxia and inhibition of mitochondrial oxidative phosphorylation, we therefore carried out studies to determine if K_v_1.5 inhibition by these stimuli was additive with respect to that induced by AMPK activators.

**Figure 5:**
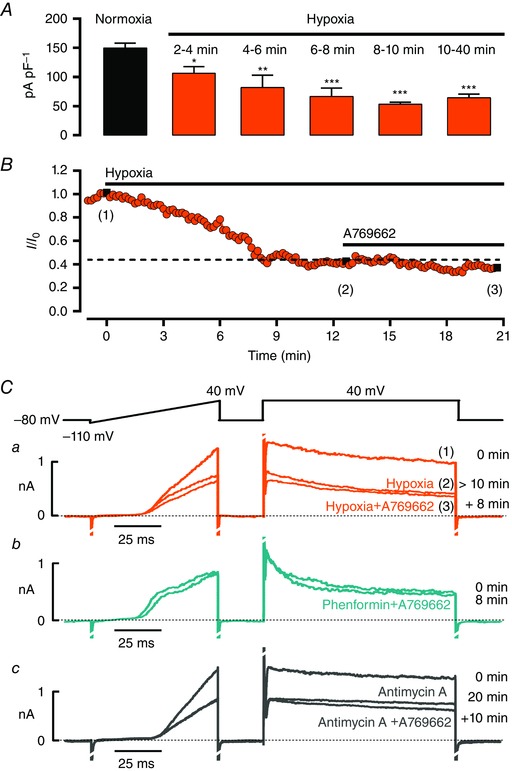
**Hypoxia and mitochondrial inhibitors attenuate K_v_1.5 currents and occlude further current inhibition by AMPK activation** *A*, bar chart showing the reduction in current density at +40 mV from myocytes superfused with a hypoxic solution (4% O_2_; *n* = 4–19). *B*, time course for reduction in K_v_ current during extracellular superfusion with hypoxic solution and the effect of subsequent addition of 100 μm A769662; measurements taken at the end of a 100 ms step pulse to +40 mV. *Ca*, example records for voltage ramp and step protocol under normoxia (1), > 10 min of hypoxia (2), after 8 min of superfusion with hypoxia + 100 μm A769662 (3). *Cb‐c*, as in *a* but representative current traces show the effect of 100 μm A769662 on myocytes pre‐incubated with 1 mm phenformin (*b*) or after 20 min extracellular application of 1 μm antimycin A (*c*). ^*^ *P* < 0.05, ^**^ *P* < 0.01 and ^***^ *P* < 0.001 *vs*. normoxia.

**Figure 6:**
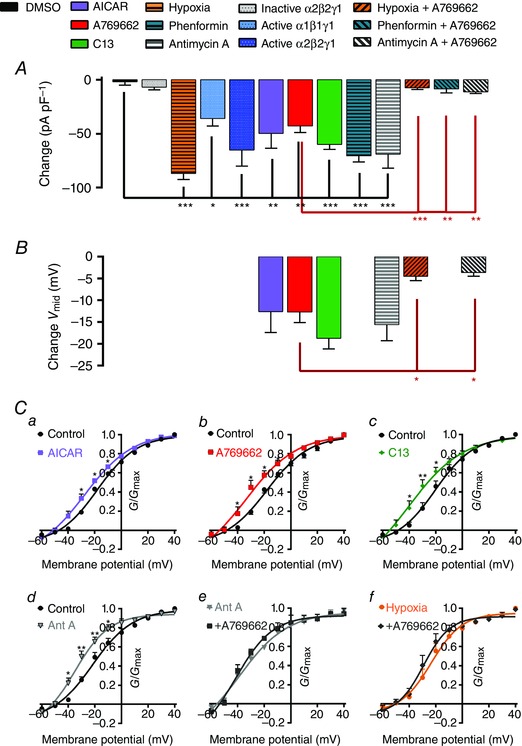
**Comparison of the change in current density and voltage–conductance relationships induced by activated AMPK and AMPK activators in pulmonary arterial myocytes** *A*, bar chart showing mean ± SEM change in current density at the end of each experimental intervention after 6–10 min of DMSO (1:1000), inactive α2β2γ2, hypoxia (∼4% O_2_), 5 U active α2β2γ2, 5 U active α1β1γ1, 1 mm AICAR, 100 μm A769662, 30 μm C13, 1 mm phenformin (2–4 h), 1 μm antimycin A, 100 μm A769662 in the presence of hypoxia (∼4% O_2_, > 10 min), phenformin and antimycin A (*n* = 3–16). *B*, similar to *A* but showing the net change in *V*_mid_ of the voltage–conductance plots for 1 mm AICAR, 100 μm A769662, 30 μm C13, 1 μm antimycin A, 100 μm A769662 in the presence of 1 μm antimycin A and 100 μm A769662 in the presence of hypoxia (∼4% O_2_, > 10 min); *n* = 3–7. *C*, voltage–conductance plots showing effects of 1 mm AICAR (*a*), 100 μm A769662 (*b*), 30 μm C13 (*c*), 1 μm antimycin A (*d*), 100 μm A769662 in the presence of 1 μm antimycin A (*e*) and 100 μm A769662 in the presence of hypoxia (*f*, ∼4% O_2_, > 10 min). Results are expressed as mean ± SEM, *n* = 3–7. ^*^ *P* < 0.05, ^**^ *P* < 0.01 and ^***^ *P* < 0.001.

Superfusion of pulmonary arterial myocytes with a hypoxic solution (∼4% O_2_, > 10 min) markedly inhibited K_v_1.5 currents, with a maximal reduction achieved after ∼10 min (Fig. 5 *A*, *B*, 150 ± 9 pA pF^–1^ during normoxia *vs*. 63 ± 6 pA pF^–1^, *n* = 14–19, after 10–40 min of hypoxia; *P* < 0.001). Most importantly, continued hypoxia markedly attenuated the degree of K_v_1.5 inhibition upon application of 100 μm A769662, which reduced current density from 53 ± 4 pA pF^–1^ during hypoxia alone to 45 ± 5 pA pF^–1^ (*n* = 6, *P* < 0.05; Figs 5 *B*, *C* and 6 *A*). As described previously, pre‐incubation of acutely isolated myocytes with 1 mm phenformin (2 h) also caused pronounced reductions in K_v_ current density, from 126 ± 17 to 55 ± 6 pA pF^–1^ at +40 mV (Fig. 1 *Ca–b*, *n* = 9–11, *P* < 0.001). Moreover, and consistent with the effects of hypoxia, pre‐incubation of cells with phenformin markedly attenuated the degree to which K_v_1.5 currents were inhibited upon application of A769662, which reduced current density from 57 ± 12 pA pF^–1^ in the presence of phenformin alone to 49 ± 10 pA pF^–1^ (Figs 5 *Cb* and 6 *A*; *n* = 4, *P* = 0.09). In this respect antimycin A was equally effective, A769662 reducing K_v_1.5 current density from 44 ± 4 pA pF^–1^ in the presence of antimycin A alone to 33 ± 4 pA pF^–1^ (Figs 5 *Cc* and 6 *A*; *n* = 3, *P* < 0.05). Therefore, K_v_1.5 inhibition by pharmacological AMPK activation is non‐additive with respect to the action of either hypoxia or respiratory poisons. We can therefore conclude that in myocytes from near‐resistance‐sized pulmonary arteries, AMPK probably acts as the primary downstream mediator of K_v_1.5 channel inhibition not only during hypoxia, but also in response to the inhibition of mitochondrial oxidative phosphorylation.

### AMPK activators induce a leftward shift in the *I–V* relationship for K_v_1.5 current activation that is occluded by hypoxia and mitochondrial inhibitors

AMPK activation not only reduced K_v_1.5 current density in pulmonary arterial myocytes throughout the *I–V* range over which K_v_1.5 currents were activated, but also induced a significant 12–14 mV hyperpolarizing shift in the activation curve (Fig. 6 *B*, *C*) analysed as *G*/*G*_max_ and fitted to a single Boltzmann function. *V*_mid_ measured: −17.9 ± 1.2 and −30.5 ± 4.5 mV in the absence and presence of AICAR, respectively; −17.4 ± 1.6 and −30.2 ± 3.5 mV in the absence and presence of A769662; −20.9 ± 2.6 and −39.7 ± 4.6 mV in the absence and presence of C13 (*n* = 5–7, *P* < 0.01). To allow for direct comparison of the maximal effect of each of these agents, we also expressed the leftward shift as the net change in *V*_mid_ (Fig. 6 *B*); Δ*V*_mid_ was −12.6 ± 4.8, −12.7 ± 2.4 and −18.7 ± 2.3 mV, respectively, for AICAR, A769662 and C13. As previously observed for current inhibition, the leftward shift in the *I–V* range for K_v_1.5 activation that was induced by AMPK activation was non‐additive with respect to the effect of hypoxia and prior inhibition of mitochondrial oxidative phosphorylation, Δ*V*_mid_ for A769662 measuring −4.5 ± 0.9 mV in the presence of hypoxia and −3.7 ± 0.6 mV in the presence of antimycin A.

**Figure 7:**
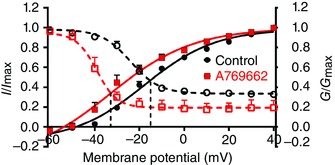
**A769662 induces a leftward shift in the activation and inactivation curves of K_v_1.5** Plot shows the voltage–conductance relationship for K_v_1.5 activation and inactivation in the absence (control, black) and presence of 100 μm A769662 (red). Activation is indicated by filled symbols and continuous lines; inactivation is indicated by open symbols and dashed lines. Data points are mean ± SEM (*n* = 3–6). Curves were obtained by fitting to the sigmoidal Boltzmann equation.

To explore further the functional significance of a leftward shift in the *I–V* relationship we assessed the voltage‐dependence of both K_v_1.5 activation and inactivation in the absence and presence A769662, and thus determined the effect of AMPK activation on the window current, i.e. the proportion of current at a given potential that is never inactivated. Figure 7 clearly shows that AMPK activation by A769662 induced a leftward shift in K_v_1.5 activation and inactivation curves and thus of the window current, lowering the threshold for activation while reducing the available non‐inactivating current.

### AMPK phosphorylates K_v_1.5 and reduces K^+^ currents carried by recombinant K_v_1.5 channels stably expressed in HEK 293 cells

**Figure 8:**
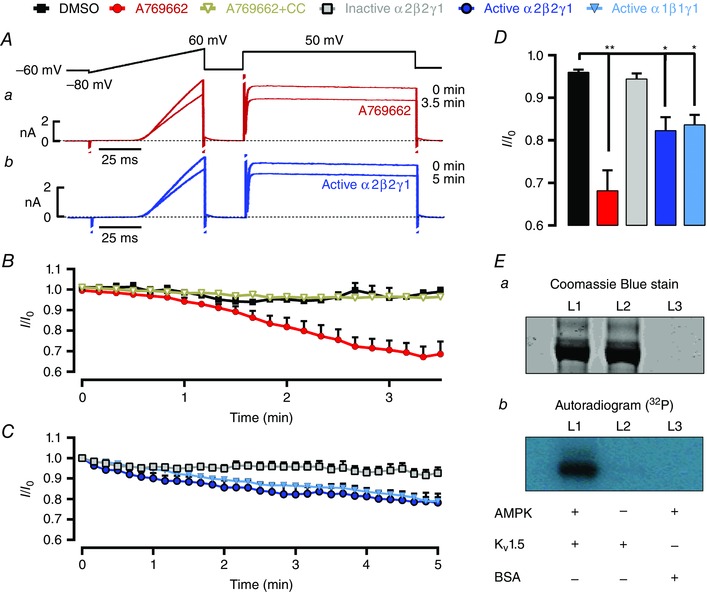
**AMPK activation phosphorylates K_v_1.5 and reduces K^+^ currents carried by recombinant K_v_1.5 channels stably expressed in HEK 293 cells** *A*, K^+^ currents carried by K_v_1.5 stably expressed in HEK 293 cells before (0 min) and 3.5 min after extracellular application of (*a*) 100 μm A769662 or 5 min following intracellular dialysis of the active AMPK heterotrimer (*b*) (α2β2γ1, thiophosphorylated, 2 U ml^−1^). *B*, time course for reduction of whole‐cell K^+^ currents during extracellular application of DMSO (1:1000, vehicle control), 100 μm A769662, the combined application of 40 μm compound C and 100 μm A769662; or *C*, intracellular dialysis of active α2β2γ1 (thiophosphorylated, 2 U ml^−1^), active α1β1γ1(thiophosphorylated, 2 U ml^−1^) or inactive α2β2γ1 (D157A mutant). *D*, bar chart showing mean ± SEM residual current at the end of each experimental intervention (3.5–5 min). Results are means ± SEM, *n* = 3–7.^*^ *P* < 0.05 and ^**^ *P* < 0.01 *vs*. control. *E*, Coomassie blue stained SDS‐PAGE (*a*) and autorad (*b*) of K_v_1.5 protein incubated with okodaic acid, 200 μm AMP, ^32^P‐ATP in the presence (L1) or absence (L2) of bacterial activated AMPK (α2β2γ1), and (L3) 1 μg of BSA treated as in L1.

To determine whether AMPK modulates K_v_1.5 channel function directly, we examined the effects of AMPK activation on human K_v_1.5 channels stably expressed in HEK 293 cells. Application of A769662 (100 μm) reduced K^+^ currents carried by recombinant human K_v_1.5 (Fig. 8 *Aa*) in a manner that was blocked by the non‐selective AMPK inhibitor compound C (40 μm, Fig. 8 *B*). Moreover, intracellular dialysis of active AMPK α2β2γ1 or α1β1γ1 heterotrimers also reduced K_v_1.5 currents, which remained unaffected in the presence of an inactive (D157A mutant) α2β2γ1 heterotrimer (Fig. 8 *Ab–C*). Like pulmonary arterial myocytes, therefore, currents carried by human K_v_1.5 expressed in HEK 293 cells were similarly inhibited both by AMPK activators and by intracellular dialysis of recombinant active AMPK heterotrimers (Fig. 8 *D*).

We also examined whether AMPK directly phosphorylates K_v_1.5, using as substrate the human protein immunoprecipitated from these HEK 293 cells. We first treated the immunoprecipitate with recombinant protein phosphatase (PP1γ) to remove endogenous phosphate groups, then phosphorylated with purified rat liver AMPK (a mixture of α1β1γ1 and α2β1γ1 isoforms) and [γ‐^32^P]ATP in the presence and absence of 200 μm AMP. The stoichiometry of phosphorylation was estimated by cutting out and counting the ^32^P‐labelled band and estimating the protein content by comparison with serum albumin standards run on the same gel. We obtained estimates of 0.57 and 0.13 moles of phosphate per mole of protein in the presence and absence of AMP (data not shown). We repeated the experiment using recombinant human α1β1γ1 and α2β2γ1 complexes expressed in bacteria, and obtained stoichiometries of 0.7 and 1.6 moles of phosphate per mole of protein respectively (Fig. 8 *E*, we did this only in the presence of AMP because, for reasons that remain unclear, the bacterially expressed complexes are much less AMP‐dependent). While we have not yet determined the number and identity of the sites phosphorylated on K_v_1.5, these results indicate that different AMPK complexes can catalyse a substantial AMP‐activated phosphorylation of K_v_1.5 in cell‐free assays.

## Discussion

The present investigation describes, for the first time, evidence that AMPK couples K_v_1.5 channel function (defined by the K_v_1.5 blocker DPO‐1) to the inhibition by hypoxia of mitochondrial metabolism in pulmonary arterial myocytes. Consistent with this proposal, inhibition by phenformin or hypoxia of the mitochondrial electron transport chain (El‐Mir *et al*. 2000; Owen *et al*. 2000) increases NAD(P)H autofluorescence (Evans *et al*. 2005), activates AMPK and inhibits K_v_ currents in pulmonary arterial myocytes. That AMPK activation may specifically regulate K_v_1.5 in response to metabolic stresses such as hypoxia gained further support from our findings that AMPK activators that are structurally distinct and have different mechanisms of action, namely A769662, AICAR and C13, all markedly inhibited K_v_ currents in pulmonary arterial myocytes. A769662, which primarily causes allosteric activation (Goransson *et al*. 2007; Scott *et al*. 2014), binds in a site located between the α and β subunits of AMPK (Xiao *et al*. 2013). AICAR (Corton *et al*. 1995) and C13 (Gomez‐Galeno *et al*. 2010) act similarly in the sense that they are both taken up into cells and converted to molecules (ZMP and C2, respectively) that bind to the γ subunit, mimicking the effects of AMP. However, C2 is a much more potent activator of AMPK than ZMP and, unlike the latter, does not affect other AMP‐sensitive enzymes such as glycogen phosphorylase or fructose‐1,6‐bisphosphatase (Hunter *et al*. 2014). Moreover, A769662 is selective for complexes containing the β1 subunit (Scott *et al*. 2008), while C2 is selective for complexes containing the α1 subunit (Hunter *et al*. 2014). These data suggest that K_v_1.5 current inhibition in pulmonary arterial myocytes may be delivered in whole or in part by AMP‐dependent activation of heterotrimers containing α1 and β1. Furthermore, inhibition of K_v_1.5 by hypoxia or by pre‐incubation with inhibitors of mitochondrial oxidative phosphorylation prevented further current reduction by AMPK activators, suggesting that AMPK may act as the primary regulator of K_v_1.5 downstream of inhibition of mitochondrial oxidative phosphorylation during hypoxia. This conclusion is also supported by previous findings that hypoxia activates AMPK (Evans *et al*. 2005) and achieves maximal AMPK phosphorylation within ∼10 min (Ibe *et al*. 2013). Crucially, given the possible off‐target effects of any pharmacological agents, intracellular dialysis of active and phosphatase‐resistant (thiophosphorylated) recombinant AMPK heterotrimers selectively inhibited K_v_ currents in pulmonary arterial myocytes, while a kinase‐dead AMPK mutant was without effect. Although our results with C13 and A769662 suggest that an endogenous α1β1‐containing complex may regulate K_v_1.5 in pulmonary arterial myocytes, our results with intracellular dialysis of α1β1γ1 and α2β2γ1 heterotrimers suggest that both are capable of regulating K_v_1.5.

It is also interesting to note that AMPK activation not only induced a leftward shift in half maximal activation of K_v_ currents, as previously reported for effects of mitochondrial inhibitors (Firth *et al*. 2008), but also a leftward shift in half maximal inactivation and thus reduced the available non‐inactivating current, i.e. the window current. The overall effect would be to lower the threshold for K_v_1.5 activation and thus increase the threshold for membrane depolarization, while reducing the opposition to membrane depolarization once initiated. These outcomes argue against a role for K_v_1.5 inhibition in the initiation, through membrane depolarisation, of acute HPV. This view is supported by the fact that compound C inhibits K_v_1.5, but has little or no effect on resting pulmonary arterial tone despite the fact that it inhibits acute HPV in a concentration‐dependent manner (Robertson *et al*. 2008). When considered together, our study therefore provides further support for the view that acute HPV is induced in a manner independent of K_v_1.5 inhibition (Dipp *et al*. 2001; Prieto‐Lloret *et al*. 2015). That aside, our findings suggest that care must be taken when assessing studies that have employed compound C to examine the role of AMPK in pulmonary vascular function, given that the marked attenuation of K_v_1.5 by compound C presents an important confounding variable with respect to investigations on myocyte proliferation and the progression of pulmonary hypertension (Ibe *et al*. 2013). Moreover in a screen of 70 protein kinases, at least 10 were inhibited by compound C more potently than AMPK (Bain *et al*. 2007). Compound C cannot, therefore, be considered to be a selective inhibitor of AMPK, a point reinforced by our findings.

**Figure 9:**
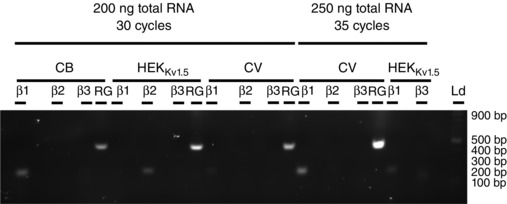
**Transcripts for all three K_v_β genes are expressed in the HEK_Kv1.5_ cell line** Gel showing RT‐PCR amplicons for K_v_β1, K_v_β2, K_v_β3 and the reference gene (RG), GAPDH, from canine brain (CB), canine ventricle (CV) and the HEK_Kv1.5_ stable line used in this study. The 500 base pair band of the ladder represents ∼1200 ng of DNA (2‐Log DNA Ladder, New England Biolabs, Ipswich, MA, USA).

Our conclusions from studies on native K_v_ currents in pulmonary arterial myocytes were confirmed by further investigations on the regulation of hK_v_1.5 stably expressed in HEK 293 cells. Native rat liver AMPK and two combinations (α1β1γ1 and α2β2γ1) of bacterially expressed human AMPK isoforms were found to incorporate near‐stoichiometric amounts of phosphate into immunoprecipitated hK_v_1.5 channels, and with rat liver AMPK this was stimulated by AMP, making it very unlikely that the phosphorylation was catalysed by a contaminating kinase. AMPK activators and recombinant heterotrimers also inhibited currents carried by recombinant K_v_1.5 channels in intact HEK 293 cells. This suggests that AMPK may directly regulate the channel protein even though K_v_β1, K_v_β2 and K_v_β3 may be expressed to varying degrees in HEK 293 cells that stably express K_v_1.5 (Fig. 9), as has been reported previously (Platoshyn *et al*. 2004).

Our results support a model in which inhibition of mitochondrial oxidative phosphorylation by hypoxia in pulmonary arterial myocytes triggers AMPK‐dependent inhibition of K_v_1.5 channels, in line with the observation that co‐expression of K_v_1.5 and AMPK reduced K_v_ current and K_v_1.5 channel abundance in the cell membrane of oocytes (Mia *et al*. 2012). Our finding that AMPK phosphorylates and inhibits K_v_1.5 is also entirely consistent with previous evidence that AMPK mediates acute HPV (Evans *et al*. 2005; Robertson *et al*. 2008), and the proposal that AMPK may also contribute to smooth muscle proliferation and the development of pulmonary arterial hypertension (Ibe *et al*. 2013; Goncharov *et al*. 2014). This is evident from the fact that down‐regulation of K_v_1.5 expression and activity is a hallmark not only of HPV but also of pulmonary hypertension (Yuan *et al*. 1998; Michelakis *et al*. 2002; Bonnet *et al*. 2006; Remillard *et al*. 2007; Burg *et al*. 2010; Morales‐Cano *et al*. 2014). This down‐regulation may lead to increased survival of smooth muscle cells due to attenuation of K^+^ channel‐dependent apoptosis (Krick *et al*. 2001; Brevnova *et al*. 2004; Moudgil *et al*. 2006), and facilitate the phenotypic switch from a contractile to a proliferative state (Cidad *et al*. 2012, 2015). Further support for this view may be taken from the finding that over‐expression of K_v_1.5 enhances apoptosis (Brevnova *et al*. 2004), while adenoviral transgene expression of K_v_1.5 *in vivo* reduces pulmonary hypertension and restores HPV (Pozeg *et al*. 2003). Therefore, it is possible that dysfunction of the mitochondrial–AMPK signalling pathway may predispose individuals to hypoxia and other forms of pulmonary arterial hypertension (Bonnet *et al*. 2006). In this respect, it is interesting to note that single nucleotide polymorphisms (SNPs) in the gene encoding K_v_1.5 predispose to pulmonary hypertension and reduce K_v_1.5 channel availability in pulmonary arterial myocytes (Remillard *et al*. 2007), raising the intriguing possibility that this may be due, at least in part, to alterations in AMPK‐dependent regulation of K_v_1.5.

AMPK phosphorylates target proteins containing a Φ(X,β)XXS/TXXXΦ (Φ, hydrophobic; β, basic) recognition motif (Hardie *et al*. 2016). The protein sequence for K_v_1.5 presents 15 serines and 4 threonines susceptible to phosphorylation by serine–threonine kinases (Blom *et al*. 1999). However, none of these represents good matches to the consensus recognition sites for AMPK (http://scansite3.mit.edu), despite the fact that our studies on ^32^P phosphorylation indicate that the immunoprecipitated channel protein might be a direct substrate for AMPK. This raises two distinct possibilities, (1) AMPK recognises non‐canonical sites within the K_v_1.5 sequence, as has been shown for other proteins (Jones *et al*. 2005; Chang *et al*. 2009; Egan *et al*. 2011); or (2) AMPK phosphorylates one or more associated protein(s), such as the regulatory β subunits. The fact that AMPK phosphorylates and regulates K_v_1.5 suggests that its effects are mediated, at least in part, independently of such interactions. Nevertheless, we cannot rule out the possibility that outcomes may be modulated by the β subunits, given that rat pulmonary arterial myocytes express K_v_β1, K_v_β2 and K_v_β3 (Platoshyn *et al*. 2006) and phosphorylation of either K_v_1.5 or regulatory K_v_β  subunits may modulate not only channel gating and inactivation kinetics (Holmes *et al*. 1996; Williams *et al*. 2002) but also the sensitivity of K_v_1.5 to regulation by K_v_β subunits (Kwak *et al*. 1999; David *et al*. 2012; Macias *et al*. 2014). It is equally plausible that AMPK‐dependent phosphorylation of K_v_1.5 in pulmonary arterial myocytes may alter K_v_α−K_v_β  interactions, sensitivity to metabolic stress, channel trafficking (Martens *et al*. 1999; Tipparaju *et al*. 2012) and/or degradation via ubiquitin ligases (Mia *et al*. 2012; Andersen *et al*. 2015). Further studies will be aimed at identifying the AMPK phosphorylation sites on K_v_1.5 and on associated β subunits.

In conclusion, we propose that AMPK couples the inhibition of mitochondrial oxidative phosphorylation to K_v_1.5 channel inhibition in pulmonary arterial myocytes, which may contribute to the regulation by AMPK of smooth muscle proliferation and thus to the development of pulmonary hypertension. In addition, AMPK‐dependent modulation of K_v_1.5 channel availability may also contribute to proliferative potential associated with other diseases, such as cancer (Bonnet *et al*. 2007; Comes *et al*. 2013; Vallejo‐Gracia *et al*. 2013).

## Additional information

### Competing interests

None of the authors have any disclosures.

### Author contributions

This manuscript was written by J.M‐S. and A.M.E. All authors contributed to the conception and design or analysis and interpretation of data, and the drafting of the article or revising it critically for important intellectual content. All authors provided final approval of the version to be published. In particular, the idea for the study was conceived by A.M.E. Tissue collection and isolation of pulmonary arterial smooth muscle cells were done by J.M‐S. J.M‐S. completed all electrophysiology on pulmonary arterial myocytes. A.M.D. completed electrophysiology on HEK293 cells. AMPK trimers were provided by D.G.H. Immunoprecipitate kinase assays and phosphorylation assays were done by F.A.R. D.F and J.E. generated the HEK293 cell line that stably expressed hK_v_1.5 and analysed the expression of K_v_β in these cells. Studies were conducted at the Centre of Integrative Physiology (University of Edinburgh, Edinburgh, UK), College of Life Sciences (University of Dundee, Dundee, UK) and Department of Anaesthesiology. Pharmacology and Therapeutics, University of British Columbia (Life Sciences Centre, Vancouver, Canada). All authors revised and approved the final version of the manuscript.

### Funding

This work was primarily funded by the Wellcome Trust (WT081195MA and WT097726) and the British Heart Foundation (RG/12/14/29885), but was also supported by the Canadian Institute for Health Research and the Heart and Stroke Foundation of Canada.
